# MCLF: Montage consistent CNN–Liquid fusion for long-term scalp EEG seizure detection

**DOI:** 10.1016/j.mex.2026.103929

**Published:** 2026-04-25

**Authors:** Ying Wang, Xuelian Zhao, Xin Yin

**Affiliations:** Zibo Central Hospital, 255020, Shandong, China

**Keywords:** Scalp EEG, Seizure detection, Multi-channel fusion, Channel serialization, Liquid neural networks, Evidence accumulation, **MCLF:** Montage consistent CNN...Liquid Fusion for seizure detection

## Abstract

Long-term scalp EEG monitoring yields hours of multi-channel recordings in which seizure-related patterns may appear only on a subset of derivations and can be obscured by transient artifacts. This work presents MCLF, a montage-consistent CNN–Liquid fusion method that implements cross-channel evidence integration as a state-based accumulation process within each epoch. Specifically, a shared 1D CNN encodes each channel into a common embedding space; embeddings are then arranged in a montage-consistent order and integrated by liquid state evolution to form an epoch-level representation for seizure scoring. A lightweight event-formation step converts the score sequence into clinically interpretable seizure events. Validation on the CHB-MIT dataset reports 100% event sensitivity with an FDR of 0.98/h and a mean latency of 2.33 s, while maintaining competitive segment-level performance relative to representative baselines. Key steps of the proposed method include:

Apply per-epoch DWT reconstruction (Db4, 5 levels) followed by z-score normalization to standardize inputs for long-term recordings.
Perform montage-consistent channel serialization and fuse the resulting channel stream via liquid state evolution for state-based evidence accumulation.
Form seizure events from epoch-level scores using MAF smoothing, thresholding, collar expansion, and event merging with validation-based parameter calibration.

## Graphical abstract

**Figure:**
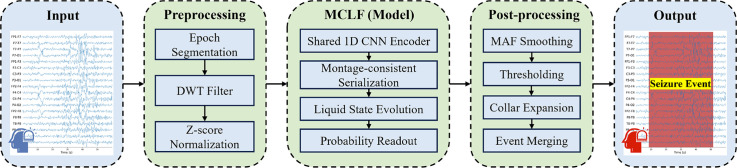

## Specifications table

## Background

Epilepsy is a common neurological disorder affecting approximately 50 million people worldwide [1]. Long-term scalp EEG monitoring is routinely used for seizure assessment, but expert visual review is labor-intensive and difficult to scale with prolonged recordings [2]. This motivates automated seizure detection systems that operate on long recordings and provide clinically actionable alarms [3], [4].

A practical constraint in long-term scalp EEG is that seizure-related changes are typically weak and spatially heterogeneous, often appearing clearly only in a subset of derivations, while artifacts frequently occur as short, high-amplitude transients confined to a small number of channels [5], [6]. For event-level detection, alarms must be formed from epoch-wise scores [7]. Therefore, the reliability of event formation depends not only on segment-wise discrimination but also on how cross-channel evidence is integrated under transient disturbances. Many modern architectures improve representation learning and temporal modeling, but channel integration is commonly implemented via implicit mixing (e.g., convolutional fusion/flattening, pooling) or one-shot reweighting, which does not explicitly regulate how evidence accumulates across channels for event formation [8], [9].

This method article describes a channel-stimulated CNN–Liquid framework that treats multi-channel fusion as an evidence accumulation process aligned with event-level detection requirements. The key idea is to (i) map each channel waveform into a common embedding space using a shared 1D CNN, (ii) serialize channels in a montage-consistent fixed order to obtain a deterministic channel stream, and (iii) integrate the stream through controlled state evolution using liquid neural dynamics [10]. This design accumulates consistent cross-channel evidence while limiting the influence of channel-local transients. The method outputs epoch-wise seizure probabilities that are subsequently converted into seizure events by a simple post-processing step.

## Method details

### Problem formulation

We formulate seizure detection as epoch-level binary classification followed by event formation. Let $X\in R^{C\times T}$ denote a preprocessed EEG epoch with *C* channels and *T* samples per channel. Let $T={\left\{\left[t_{s}^{i},t_{e}^{i}\right]\right\}}_{i=1}^{N}$ be the set of annotated seizure intervals in a recording. The label *y* ∈ {0, 1} indicates whether the time span of **X** has a non-empty intersection with any interval in T. We learn an end-to-end mapping *F*( · ) that outputs an estimated seizure probability $\hat{p}=F\left(X\right)\in \left[0,1\right]$.

### Methodology

**Fig. 1:**
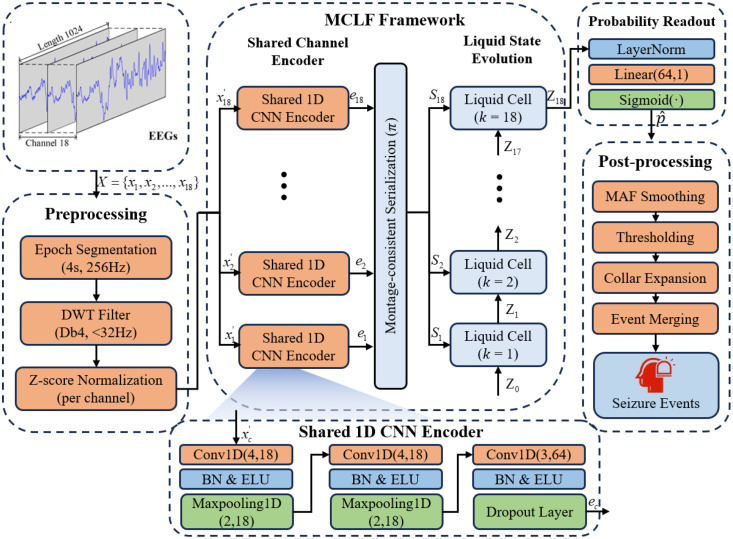
MCLF framework for long-term scalp EEG seizure detection.

In long-term scalp EEG, channel quality can fluctuate over time, while seizure-related patterns may be weak and unevenly expressed across derivations. Accordingly, we design the fusion module to perform controlled cross-channel evidence accumulation, reducing the influence of channel-local disturbances on epoch-wise scoring. Fig. 1 provides an overview of MCLF, including (i) epoch-wise preprocessing, (ii) channel-stimulated CNN–Liquid modeling with montage-consistent channel serialization and liquid state evolution, and (iii) post-processing for event formation.

#### Data preprocessing

We partition each recording into fixed-length epochs of 4 s at $f_{s}=256$ Hz, yielding T=1024 samples per channel. Each epoch is denoted by $X={\left[x_{1},\ldots ,x_{C}\right]}^{⊤}\in R^{C\times T}$, where $x_{c}\in R^{T}$ is the raw waveform of the *c*th channel. Scalp EEG frequently exhibits transient high-frequency bursts, often associated with EMG contamination. These bursts introduce short-lived, channel-local perturbations that can degrade epoch-wise scoring and downstream event formation. To suppress such bursts while remaining consistent with epoch-wise modeling and avoiding cross-epoch boundary effects, we apply *segment-level* wavelet filtering independently to each epoch.

Specifically, we perform a 5-level DWT (Db4) [11] and reconstruct the low-frequency signal for each channel as

(1) $${\hat{x}}_{c}=\text{IDWT}(cA_{5}+cD_{5}+cD_{4}+cD_{3}),\;c=1,\ldots ,C,$$

which retains components approximately within 0–32 Hz and attenuates higher-frequency content that is more likely to be contaminated by EMG activity. This filtering reduces the impact of transient high-frequency bursts on epoch-level scoring, facilitating more reliable event formation.

Channel statistics also vary substantially across sessions and subjects due to differences in electrode impedance, montage configuration, and low-frequency drift. Such variability complicates optimization and reduces the robustness of fixed-threshold event formation across recordings. Accordingly, each reconstructed channel is normalized within the epoch using z-score normalization:

(2) $$x_{c}^{\prime }=\frac{{\hat{x}}_{c}-\mu_{c}}{\sigma_{c}+\epsilon },\;c=1,\ldots ,C,$$

where *μ_c_* and *σ_c_* are computed over the samples of channel *c* in the current epoch, and ϵ is a small constant for numerical stability. The normalized epoch $X^{\prime }={\left[x_{1}^{\prime },\ldots ,x_{C}^{\prime }\right]}^{⊤}$ is then fed into the model.

#### Channel-stimulated CNN–liquid model

The model integrates multi-channel evidence through a two-stage design. First, a shared channel encoder extracts channel-wise features in a common latent space. Second, a liquid dynamics module integrates these features along the channel dimension via controlled state evolution to accumulate cross-channel evidence.

**Shared channel encoder.** Seizure-related cues in long-term scalp EEG often manifest as brief and localized waveform changes. We therefore employ a shared 1D CNN encoder to extract local temporal patterns from each channel and map the waveform into a compact embedding prior to cross-channel fusion. Given the *c*th channel waveform $x_{c}^{\prime }\in R^{T}$, the encoder outputs

(3) $$e_{c}=f_{\theta }\left(x_{c}^{\prime }\right)\in R^{d},\;c=1,\ldots ,C,$$

where *d* denotes the embedding dimension. The same encoder is applied to all channels, which enforces a consistent feature space and avoids channel-specific parameters, facilitating subsequent state-based evidence accumulation across channels.

The encoder consists of three convolutional blocks. The first two blocks use Conv1D layers with kernel size 4 and 18 output channels, each followed by batch normalization, ELU activation, and MaxPooling1D with pool size 2. The third block uses Conv1D with kernel size 3 and 64 output channels, followed by batch normalization and ELU. The encoder configuration is summarized in Table 1.

**Table 1:** Shared 1D CNN channel encoder.

Layer	Out-ch	Kernel/Pool	Stride	Padding	Output shape
Input	1	–	–	–	1 × 1024
Conv1D + BN + ELU	18	4	2	same	18 × 512
MaxPooling1D	–	2	2	–	18 × 256
Conv1D + BN + ELU	18	4	2	same	18 × 128
MaxPooling1D	–	2	2	–	18 × 64
Conv1D + BN + ELU	64	3	2	same	64 × 32
GlobalAvgPool1D	–	–	–	–	64 × 1

**Channel serialization and liquid aggregation.** Seizure-related activity in scalp EEG typically appears as structured spatial patterns across derivations rather than as independent observations from individual channels. To make cross-channel evidence accumulation physiologically interpretable, we adopt a fixed montage-consistent channel order (Table 2) comprising anatomically contiguous bipolar chains arranged from anterior to posterior. This ordering is shared across all subjects and recordings, enabling deterministic and comparable cross-channel fusion.

**Table 2:** Montage-consistent order of 18 bipolar derivations used for channel serialization.

Chain	Bipolar derivations
Left lateral	FP1-F7, F7-T7, T7-P7, P7-O1
Left parasagittal	FP1-F3, F3-C3, C3-P3, P3-O1
Right parasagittal	FP2-F4, F4-C4, C4-P4, P4-O2
Right lateral	FP2-F8, F8-T8, T8-P8, P8-O2
Midline	FZ-CZ, CZ-PZ

Given the channel embeddings ${\left\{e_{c}\right\}}_{c=1}^{C}$, we define an ordered within-epoch stimulus sequence

(4) $$S_{k}=e_{\pi (k)},\;k=1,\ldots ,C,$$

where *π* denotes the permutation induced by the predefined channel order and *k* indexes pseudo-time along the ordered channel sequence.

Let $Z_{k}\in R^{d}$ denote the latent fusion state after integrating the first *k* ordered channel inputs, with initialization $Z_{0}=0$. We adopt an LNN-style gated update to accumulate cross-channel evidence sequentially:

(5) $$Z_{k}=\frac{Z_{k-1}+\Delta \;f\left(Z_{k-1},S_{k};\theta \right)⊙A}{1+\Delta \left(\frac{1}{\tau }+f\left(Z_{k-1},S_{k};\theta \right)\right)},$$

where $\tau \in R^{d}$ is a learnable positive time-constant vector, $A\in R^{d}$ is a learnable scaling vector, and ⊙ denotes element-wise multiplication. This update implements controlled evidence accumulation: previously integrated information is adaptively damped, while seizure-related patterns that are expressed consistently across multiple channels are reinforced through repeated gated injections.

The modulation function is instantiated as a bounded gate:

(6) $$f\left(Z_{k-1},S_{k};\theta \right)=\sigma \;(\text{mish}(W_{z}Z_{k-1}+W_{s}S_{k}+b)),$$

where $W_{z},W_{s}\in R^{d\times d}$ and $b\in R^{d}$ are learnable parameters. The sigmoid function constrains the gate to (0, 1)^*d*^, which helps keep the accumulation dynamics well behaved.

For each channel input **S**_*k*_, we perform *U* unfolded solver sub-steps. One channel step corresponds to a unit pseudo-time interval, and we set Δ=1/U. Specifically,

(7) $$\begin{array}{cc} Z_{k}^{\left(0\right)} & =Z_{k-1}, \end{array}$$

(8) $$\begin{array}{cc} Z_{k}^{\left(u\right)} & =\text{LNNUpdate}\;\left(Z_{k}^{\left(u-1\right)},\;S_{k};\;\Delta \right),\;u=1,\ldots ,U, \end{array}$$

and the final state is taken as $Z_{k}=Z_{k}^{\left(U\right)}$. This unfolding increases fusion capacity while keeping the channel sequence length fixed.

After processing all *C* channels, we obtain the epoch-level representation **Z**_*C*_ and compute the seizure probability by

(9) $$\hat{p}=\sigma (w^{⊤}\text{LN}\left(Z_{C}\right)+b),$$

where LN( · ) denotes LayerNorm, and $w\in R^{d}$ and b∈R are learnable parameters.

#### Post-processing

Post-processing converts epoch-wise probabilities into event-level detections for long recordings. The procedure consists of score smoothing, thresholding, collar expansion, and event merging.

Let ${\hat{p}}_{t}$ denote the model output for the *t*th epoch. We apply a centered moving average filter (MAF) of odd length L=2N+1 to obtain a smoothed score sequence:

(10) $$Y_{t}=\frac{1}{2N+1}\sum_{n=-N}^{N}{\hat{p}}_{t+n},$$

where *Y_t_* is the output after MAF and 2N+1 is the MAF length. For indices near the boundaries, we compute Eq. (10) on the valid range and normalize by the number of valid terms. We then obtain binary epoch labels by thresholding:

(11) $${\hat{y}}_{t}=I\left(Y_{t}\geq \text{th}\right),$$

where I(·) denotes the indicator function and th is the selected threshold.

MAF suppresses short-lived fluctuations but also attenuates sharp boundary transitions. To compensate boundary attenuation after thresholding, we apply a collar operation that expands each detected positive segment by *r* epochs on both sides:

(12) $$r=N=\left\lfloor \frac{L-1}{2}\right\rfloor .$$

**Fig. 2:**
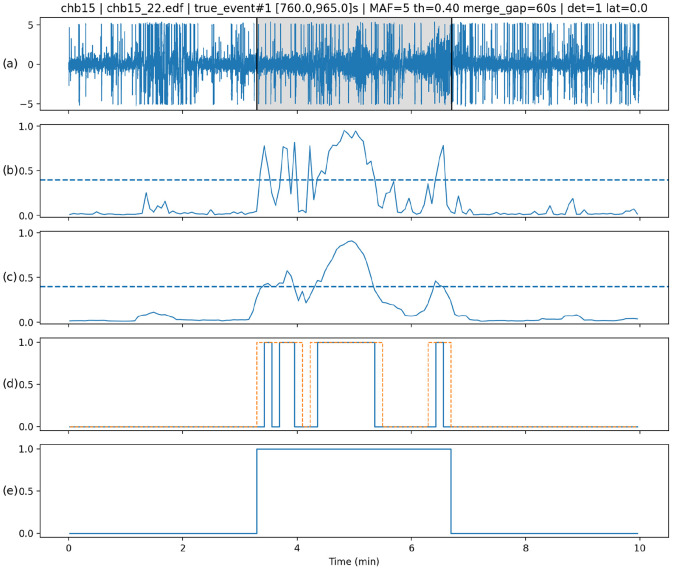
Post-processing procedure for event formation. The shaded/boxed region corresponds to the seizure interval annotated by experts, and the horizontal dashed line indicates the selected threshold. (a) EEG excerpt. (b) Raw epoch-wise probabilities ${\hat{p}}_{t}$ produced by the network. (c) Smoothed scores *Y_t_* after applying the centered MAF. (d) Binary decisions after thresholding. (e) Final event labels after collar expansion and event merging.

Finally, two detected events whose gap is no larger than *γ* seconds are merged into one event. The gap is measured in seconds by multiplying the number of intervening epochs by the epoch duration. Fig. 2 illustrates the post-processing procedure.

## Method validation

### Dataset and annotations

We evaluate the proposed method on the public CHB-MIT scalp EEG dataset, which provides long-term pediatric EEG recordings in EDF format with expert-annotated seizure onset/offset intervals [12]. All signals are sampled at 256 Hz using the international 10–20 system. Seizure annotations are represented as time intervals $T=\{\left[t_{s}^{i},t_{e}^{i}\right]\}$ (in seconds) and are used for epoch labeling, event matching, and latency computation.

To obtain a consistent input space across recordings, we use a fixed set of 18 common bipolar derivations (Table 2). EDF channels are matched and aligned by channel-name rules; any recording missing at least one required derivation is excluded rather than imputed.

To complement the CHB-MIT evaluation, we further introduced the TUH EEG Event Corpus (TUEV) for an external robustness analysis. TUEV is a subset of the TUH EEG Corpus and contains six annotated event categories: spike and slow wave (spsw), generalized periodic epileptiform discharge (gped), periodic lateralized epileptiform discharge (pled), eye movement (eyem), artifact (artf), and background (bckg).

*Epoching and sampling* Continuous EEG is segmented into fixed-length epochs of 4.0 s at $f_{s}=256$ Hz (T=1024 samples per channel). To improve coverage of seizure epochs during training while controlling redundancy in non-seizure data, we use an asymmetric stride: 2 s stride (50% overlap) within seizure intervals and 4 s stride (no overlap) outside seizures. Class imbalance is controlled by randomly downsampling non-seizure epochs with a maximum negative-to-positive ratio of 8.

### Experimental setup

*Computing environment* All experiments are conducted on a workstation equipped with an NVIDIA GeForce RTX 4090 GPU (24 GB), a 13th Gen Intel(R) Core(TM) i9-13900K CPU (3.00 GHz), and 32 GB RAM.

*Training protocol* In the CHB-MIT dataset, each subject’s long-term recording is provided as multiple EDF files. For each subject, EDF files are first divided into training, validation, and test subsets at the file level before epoch extraction. A global model is then trained using the pooled training files from all subjects and evaluated separately on the test files of each subject. This protocol avoids training/test leakage at the epoch level, since epoch extraction is performed only after the EDF-level split.

We train the model using Adam with learning rate 0.01 and batch size 256 for up to 80 epochs. Early stopping is applied based on validation loss (patience = 15); otherwise, training loss is used for model selection. Unless stated otherwise, we use C=18, T=1024, embedding dimension d=64, unfolding steps U=3, and dropout rate 0.6.

For a fair comparison, all baseline models were reproduced and evaluated under the same experimental setting as the proposed method. In this way, the reported performance differences mainly reflect differences in model architecture rather than inconsistencies in data preparation or evaluation settings.

*Post-processing calibration* Event formation uses MAF smoothing, thresholding, collar expansion, and event merging. We select the MAF length from {3, 5, 7, 9} and the threshold th from a discrete grid over [0.1,0.9] using the validation epochs, with segment-level balanced accuracy as the selection criterion. The collar radius is derived from the MAF length as k=⌊(MAF−1)/2⌋. Adjacent detected events are merged if the inter-event gap is no larger than 60 s.

*External robustness analysis on TUEV* For the external robustness analysis on TUEV, we treated spsw as the positive class and artf, eyem, and bckg as negative classes. Referential channels in TUEV were first transformed into the required bipolar derivations and then reordered according to the same montage-consistent channel sequence used in the main CHB-MIT experiments. All models were trained on CHB-MIT following the protocol described above and were subsequently evaluated on the TUEV-based external test set without retraining.

### Evaluation metrics

*Segment-based metrics* At the epoch level, predictions are compared with epoch labels to obtain TP, TN, FP, and FN. We report segment sensitivity, specificity, and accuracy:

(13) $$\begin{array}{cc} \text{SegSens} & =\frac{\text{TP}}{\text{TP}+\text{FN}}, \end{array}$$

(14) $$\begin{array}{cc} \text{SegSpec} & =\frac{\text{TN}}{\text{TN}+\text{FP}}, \end{array}$$

(15) $$\begin{array}{cc} \text{SegAcc} & =\frac{\text{TP}+\text{TN}}{\text{TP}+\text{TN}+\text{FP}+\text{FN}}. \end{array}$$

*Event-based metrics* A ground-truth seizure event [*t_s_, t_e_*] is counted as detected if it has a non-zero temporal overlap with at least one predicted event interval. Event sensitivity is defined as

(16) $${\text{Sens}}_{\text{event}}=100\times \frac{\#\text{detected}\;\text{ground-truth}\;\text{events}}{\#\text{ground-truth}\;\text{events}}.$$

Since this matching rule only requires non-zero overlap between predicted and annotated events, successful event matching can be achieved relatively easily once a predicted event partially overlaps a ground-truth seizure. Therefore, to avoid an overly optimistic interpretation of event sensitivity under this criterion, a more complete evaluation of event-level performance should additionally consider false detection rate and latency. The false detection rate per hour is

(17) $$\text{FDR}\left(/h\right)=\frac{\#\text{false}\;\text{detected}\;\text{events}}{\text{recording}\;\text{duration}\;\text{(hours)}}.$$

For each detected ground-truth event, latency is computed as

(18) $$\text{Latency}=\max\;\left(0,\;t_{\text{onset}}^{\text{pred}}-t_{\text{onset}}^{\text{gt}}\right),$$

where $t_{\text{onset}}^{\text{pred}}$ is the earliest onset time among predicted events that overlap the ground-truth event and $t_{\text{onset}}^{\text{gt}}$ is the annotated onset time.

For the external TUEV experiment, we additionally report the positive prediction rates on the artf, eyem, and bckg subsets, denoted as ARTF, EYEM, and BCKG, respectively. These values indicate the proportions of samples in each subset that are predicted as positive, and lower values correspond to better robustness against artifact-related and non-epileptiform confounding patterns.

### Results analysis

Table 3 reports per-case results on CHB-MIT, including both epoch-wise (segment-level) and event-level metrics after event formation.

**Table 3:** Per-case results on CHB-MIT. Segment-level metrics are computed at the epoch level, and event-level metrics are computed after event formation. The **Total** row reports summed Test(h), TestSeiz, and Detections, while all other metrics are averaged across cases.

Case	Test(h)	TestSeiz	Det.	Segment-level			Event-level		
				SegSens(%)	SegSpec(%)	SegAcc(%)	Sens(%)	FDR(/h)	Latency(s)
chb01	37.55	4	4	100.00	99.70	99.70	100.00	0.29	0.00
chb02	33.27	1	1	100.00	99.18	99.19	100.00	0.30	0.00
chb03	36.00	5	5	100.00	99.20	99.20	100.00	0.45	0.00
chb04	149.42	2	2	84.21	96.64	96.63	100.00	0.94	3.00
chb05	36.00	2	2	96.36	99.03	99.02	100.00	0.89	4.00
chb07	59.05	1	1	100.00	96.65	96.65	100.00	0.97	0.00
chb08	18.01	3	3	80.50	99.44	99.25	100.00	0.28	0.00
chb09	62.29	1	1	100.00	98.36	98.36	100.00	0.75	0.00
chb10	42.01	3	3	96.36	99.98	99.97	100.00	0.00	0.33
chb11	33.00	1	1	100.00	99.51	99.51	100.00	0.58	0.00
chb13	28.00	5	5	93.15	97.89	97.87	100.00	1.90	0.00
chb14	20.00	1	1	63.64	97.85	97.83	100.00	1.80	0.00
chb15	37.01	11	11	93.33	98.32	98.27	100.00	0.79	1.45
chb17	19.00	1	1	100.00	88.59	88.60	100.00	6.48	0.00
chb18	32.00	2	2	100.00	97.62	97.63	100.00	0.56	0.00
chb19	27.93	1	1	100.00	99.05	99.05	100.00	0.61	0.00
chb20	24.60	3	3	92.86	97.79	97.78	100.00	1.34	1.67
chb21	30.83	2	2	67.86	99.72	99.69	100.00	0.23	10.50
chb22	29.00	1	1	100.00	99.82	99.82	100.00	0.17	0.00
chb23	19.68	1	1	100.00	99.79	99.79	100.00	0.20	0.00
chb24	19.30	14	14	53.95	98.52	97.97	100.00	1.05	28.00

**Total**	**793.95**	**65**	**65**	**91.53**	**98.22**	**98.18**	**100.00**	**0.98**	**2.33**

**Segment-level performance.** Across cases, the averaged segment sensitivity, specificity, and accuracy are 91.53%, 98.22%, and 98.18%, respectively. Segment specificity remains high for most subjects, indicating that non-seizure epochs are generally well separated from seizure epochs in the learned representation. The main variability appears in segment sensitivity: several cases (e.g., chb14, chb21, and chb24) exhibit reduced SegSens, suggesting that seizure-related patterns in these recordings are harder to capture at the epoch level, potentially due to weaker manifestations, spatial sparsity across channels, or boundary ambiguity.

**Event-level performance.** Under the overlap-based event-matching criterion described above, all 65 annotated events were detected, corresponding to an event sensitivity of 100.00% (65/65; 95% exact binomial CI: 94.48%–100.00%). The averaged false detection rate was 0.98/h and the averaged latency was 2.33 s, while both metrics still exhibited clear case-dependent variability. In particular, chb17 showed a higher FDR (6.48/h), and a few cases (e.g., chb21 and chb24) had larger onset latency, reflecting that event formation can still be affected by transient disturbances and boundary attenuation under smoothing and thresholding. Therefore, although all annotated events were detected under the current overlap-based criterion, the accompanying FDR and latency values indicate that event-level detection quality was not uniformly perfect across recordings. Overall, these results suggest that while epoch-wise sensitivity varies across subjects, the event formation pipeline can recover event-level detections by integrating evidence over time under the present evaluation setting.

### Sensitivity analysis of post-processing parameters

Tables 4 and 5 summarize the sensitivity of the proposed framework to the main post-processing parameters, namely the MAF window length and the decision threshold.

**Table 4:** Sensitivity analysis of the MAF window length on the CHB-MIT dataset. The decision threshold is fixed at 0.5.

MAF	Segment-based			Event-based		
	Accuracy(%)	Sensitivity(%)	Specificity(%)	Sensitivity(%)	FDR(/h)	Latency(s)
3	98.18	**91.53**	98.22	**100.00**	0.98	**2.33**
5	98.03	88.06	98.26	96.80	0.90	2.64
7	98.37	87.77	98.57	95.24	0.58	2.72
9	**98.40**	86.33	**98.61**	91.63	**0.41**	26.34

**Table 5:** Sensitivity analysis of the decision threshold on the CHB-MIT dataset. The MAF window length is fixed at 3.

Threshold	Segment-based			Event-based		
	Accuracy(%)	Sensitivity(%)	Specificity(%)	Sensitivity(%)	FDR(/h)	Latency(s)
0.3	96.94	**92.48**	96.95	**100.00**	2.03	**0.85**
0.4	97.60	92.05	97.62	**100.00**	1.45	1.55
0.5	98.18	91.53	98.22	**100.00**	0.98	2.33
0.6	98.42	84.18	98.72	97.62	0.92	3.75
0.7	**98.92**	76.67	**99.21**	97.19	**0.78**	6.14

As the MAF window increased from 3 to 9, event sensitivity decreased from 100.00% to 91.63%, and segment sensitivity decreased from 91.53% to 86.33%. Meanwhile, FDR was gradually reduced from 0.98/h to 0.41/h, and specificity showed a slight increase. This indicates that stronger temporal smoothing suppresses false detections but also weakens or delays seizure-related responses. In particular, MAF=9 resulted in a substantial increase in latency to 26.34 s, suggesting that overly aggressive smoothing is unfavorable for timely event detection.

A similar trade-off was observed for the decision threshold. Lower thresholds preserved high event sensitivity and shorter latency, but at the cost of more false detections and lower specificity. In contrast, higher thresholds improved specificity and reduced false alarms overall, but led to lower segment sensitivity and longer detection latency. Among the tested settings, MAF=3 and threshold=0.5 provided the best balance between event sensitivity, false detection rate, latency, and segment-level performance.

### Sensitivity analysis under different event-matching criteria

To characterize the dependence of event-level results on the matching definition, we further evaluated the method under different minimum overlap requirements between predicted and annotated events (Table 6). With the minimum overlap set to 0 s, 15 s, 30 s, and 60 s, the averaged event sensitivity was 100.00%, 99.05%, 93.81%, and 56.37%, respectively.

**Table 6:** Sensitivity analysis under different event-matching criteria on the CHB-MIT dataset. The original setting (0 s) uses the non-zero-overlap rule, whereas stricter settings require the overlap duration between predicted and annotated events to be at least 15 s, 30 s, or 60 s. All values are averaged across the evaluated CHB-MIT cases.

Minimum overlap	Segment-based			Event-based		
	Accuracy(%)	Sensitivity(%)	Specificity(%)	Sensitivity(%)	FDR(/h)	Latency(s)
0 s	98.18	91.53	98.22	100.00	0.98	2.33
15 s	98.18	91.53	98.22	99.05	1.02	2.37
30 s	98.18	91.53	98.22	93.81	1.01	7.14
60 s	98.18	91.53	98.22	56.37	1.09	9.82

The results remained highly stable under the 15 s criterion, with only minor changes in FDR and latency (1.02/h and 2.37 s, respectively). By contrast, larger minimum-overlap requirements of 30 s and 60 s led to progressively lower event sensitivity and larger latency values. This indicates that many predicted events were already aligned with the annotated seizure intervals, but increasingly strict overlap requirements imposed a more conservative definition of successful event detection.

As expected, the segment-level metrics were identical across all settings, since only the event-level matching rule was varied. These observations indicate that the main event-level findings are stable under moderate changes in the matching criterion, while very long required overlap durations substantially tighten the definition of a matched seizure event.

### Feature visualization

To qualitatively examine how the proposed pipeline shapes the feature space, we visualize representations at three stages: (i) raw epoch features, (ii) features after preprocessing, and (iii) embeddings produced by MCLF. For each stage, we apply the same t-SNE setting to project high-dimensional features into two dimensions, and color points by their epoch labels (purple: non-seizure; yellow: seizure).

**Fig. 3:**
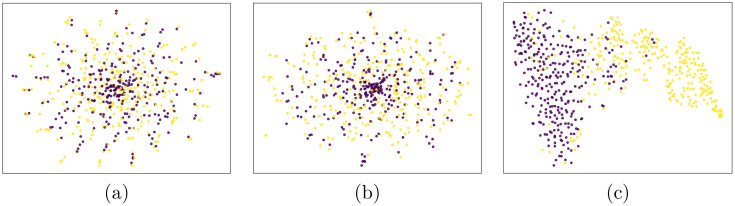
Qualitative two-dimensional visualization of features at different stages using t-SNE. (a) Raw features. (b) Features after preprocessing. (c) Model embeddings produced by MCLF.

As shown in Fig. 3(a), the raw features exhibit a highly dispersed distribution with substantial visual overlap between seizure and non-seizure epochs. After preprocessing (Fig. 3(b)), non-seizure samples appear more concentrated around the central region, whereas seizure samples remain relatively scattered. This qualitative trend is consistent with the role of preprocessing in long-term scalp EEG: segment-level DWT reconstruction suppresses high-frequency contamination and reduces short-lived perturbations, while per-epoch z-score normalization aligns channel-wise amplitude scales across sessions and subjects. Accordingly, the dominant background EEG appears more compact in the projected space, whereas seizure-related epochs remain heterogeneous due to variability in spatial manifestation and morphology across channels and subjects.

After model processing (Fig. 3(c)), the learned embeddings show a more structured visual organization, with reduced apparent overlap between the two classes. Non-seizure samples form a relatively coherent region, while seizure samples occupy a more distinct area with structured variation. These visual patterns are consistent with the intended role of MCLF in transforming preprocessed signals into embeddings that better capture seizure-relevant information under cross-channel evidence accumulation.

### Ablation study

Table 7 reports the ablation results of the main architectural components in MCLF on the CHB-MIT dataset, including DWT preprocessing, montage-consistent channel ordering, and liquid fusion.

**Table 7:** Ablation study of the main components in MCLF on the CHB-MIT dataset.

Model	Segment-based			Event-based		
	Accuracy(%)	Sensitivity(%)	Specificity(%)	Sensitivity(%)	FDR(/h)	Latency(s)
Full MCLF	**98.18**	**91.53**	**98.22**	**100.00**	**0.98**	**2.33**
w/o DWT	97.46	90.70	97.58	96.92	1.24	2.61
w/o montage consistency	97.29	90.21	97.44	95.38	1.33	2.86
w/o liquid fusion	97.08	89.84	97.31	93.85	1.43	3.12

Removing any of these components led to consistent degradation in both segment-level and event-level performance. In particular, the full MCLF achieved the best overall results, with an event sensitivity of 100.00%, an FDR of 0.98/h, a latency of 2.33 s, and segment-level metrics of 91.53% SegSens, 98.22% SegSpec, and 98.18% SegAcc.

When DWT preprocessing was removed, event sensitivity decreased to 96.92%, while FDR and latency increased to 1.24/h and 2.61 s, respectively. This indicates that the frequency-selective preprocessing step contributes to suppressing nuisance fluctuations and improving the separability of seizure-related epochs.

When montage consistency was removed, performance further decreased, with event sensitivity dropping to 95.38% and FDR rising to 1.33/h. This suggests that preserving a consistent channel ordering benefits the sequential fusion process by providing a more stable cross-channel structural prior.

The largest degradation was observed when liquid fusion was removed. In this case, event sensitivity fell to 93.85%, FDR increased to 1.43/h, latency rose to 3.12 s, and all segment-level metrics were also reduced. This result indicates that liquid fusion is the most influential component in the proposed framework, supporting its role in cross-channel evidence accumulation and temporally coherent event formation.

Overall, the ablation results show that DWT preprocessing, montage-consistent ordering, and liquid fusion all contribute positively to the final performance, while the complete MCLF yields the best balance between sensitivity, false detections, latency, and segment-level discrimination.

### Comparison with baseline methods

Table 8 compares MCLF with representative deep learning baselines on the CHB-MIT dataset, including recurrent (Bi-GRU) [13], convolution–recurrent (STFT+CNN–LSTM) [14], temporal convolution–recurrent (TCN–BiLSTM) [16], and convolution–Transformer (CNN-Transformer and CNN–Informer) models [15], [17]. All methods are evaluated using the same segment-based and event-based criteria.

**Table 8:** Comparison of different methods on the CHB-MIT dataset. “–” indicates the metric is not reported in the corresponding reference.

Author	Method	Segment-based			Event-based		
		Accuracy(%)	Sensitivity(%)	Specificity(%)	Sensitivity(%)	FDR(/h)	Latency(s)
Zhang et al. [13] (2022)	Bi-GRU	96.18	83.40	96.22	90.41	1.71	2.93
Zhou et al. [14] (2023)	STFT+CNN-LSTM	96.11	82.15	96.19	90.52	2.51	6.78
Zhou et al. [15] (2023)	CNN-Transformer	96.61	86.03	96.65	92.22	1.34	3.66
Dong et al. [16] (2024)	TCN-Bi-LSTM	96.63	85.99	96.69	92.18	1.37	4.46
Li et al. [17] (2025)	CNN-Informer	97.17	88.02	97.23	94.87	1.17	3.31

Our work	MCLF	**98.18**	**91.53**	**98.22**	**100.00**	**0.98**	**2.33**

**Segment-based results.** MCLF achieves an epoch-wise accuracy of 98.18% with 98.22% specificity and 91.53% sensitivity. Compared with the strongest baseline in segment sensitivity (CNN-Informer, 88.02%), MCLF improves SegSens by 3.51 percentage points, while also improving accuracy and specificity. Relative to other baselines, MCLF provides consistent gains in segment sensitivity, such as +5.54 points over TCN-Bi-LSTM and +9.38 points over Bi-GRU, indicating more effective discrimination of seizure epochs under long-term recordings.

**Event-based results.** After converting epoch-wise probabilities to events via post-processing, MCLF reaches 100.00% event sensitivity with an FDR of 0.98/h and a latency of 2.33 s. In comparison, the best baseline event sensitivity is 94.87% (CNN-Informer), and MCLF reduces FDR relative to all baselines (e.g., from 1.17/h to 0.98/h compared with CNN-Informer, and from 1.37/h to 0.98/h compared with TCN-Bi-LSTM). Latency is also reduced relative to all baselines in Table 8, suggesting that MCLF supports timely detection once event formation is applied.

Overall, these results indicate that montage-consistent channel serialization with liquid state evolution improves both epoch-wise discrimination and event-level detection under the CHB-MIT evaluation setting.

### External robustness analysis on artifact-labeled EEG events

Among all compared methods, MCLF achieved the best overall performance, with a SegAcc of 96.41%, a SegSens of 84.66%, and a SegSpec of 96.71% (Table 9). More importantly, MCLF yielded the lowest positive prediction rates on the artf, eyem, and bckg subsets, namely 1.91%, 0.30%, and 3.49%, respectively. Compared with the strongest baseline CNN-Informer, MCLF improved SegAcc from 92.60% to 96.41%, SegSens from 76.80% to 84.66%, and SegSpec from 93.00% to 96.71%, while further reducing the confusion rates on all three non-seizure subsets.

**Table 9:** External robustness analysis on TUEV. In this experiment, ARTF, EYEM, and BCKG denote the positive prediction rates on the corresponding subsets. Lower values indicate better robustness to non-seizure confounders.

Method	SegAcc(%)	SegSens(%)	SegSpec(%)	ARTF(%)	EYEM(%)	BCKG(%)
Bi-GRU	87.58	67.20	88.10	9.80	3.20	12.60
STFT+CNN-LSTM	83.36	62.10	83.90	10.90	4.10	15.20
CNN-Transformer	76.48	58.10	76.95	14.88	6.99	24.24
TCN-BiLSTM	89.59	69.80	90.10	6.80	2.10	8.60
CNN-Informer	92.60	76.80	93.00	4.20	1.10	5.40

Our work	**96.41**	**84.66**	**96.71**	**1.91**	**0.30**	**3.49**

**Table:** 

**Subject area**	Biomedical signal processing; clinical neurophysiology
**More specific subject area**	Long-term scalp EEG seizure detection
**Method name**	**MCLF**: **M**ontage consistent **C**NN–**L**iquid **F**usion for seizure detection
**Name and reference of original method**	N/A
**Resource availability**	Data: public long-term scalp EEG datasets https://physionet.org/content/chbmit/1.0.0 Code: https://github.com/Yinxin111/MCLF.

These results suggest that the proposed channel-wise evidence accumulation strategy is less likely to assign positive epileptiform decisions to artifact-related or non-epileptiform background events.

## Limitations

The proposed method assumes a montage-consistent fixed channel order. When montage configurations differ across datasets, an explicit channel mapping step is required before applying the model. Post-processing parameters, including the MAF length, decision threshold, and event merging gap, may need to be re-calibrated for new recording conditions and different seizure prevalence. Event-level evaluation in this study uses an overlap-based matching rule. Using alternative matching criteria, such as tolerance windows around onset and offset, can change the absolute values of reported metrics.

## Ethics statements

This study uses publicly available, de-identified EEG datasets. No new data collection involving human participants was performed.

## CRediT authorship contribution statement

**Ying Wang:** Conceptualization, Methodology, Software, Data curation, Formal analysis, Visualization, Writing – original draft. **Xuelian Zhao:** Methodology, Software, Validation, Formal analysis, Visualization, Writing – original draft. **Xin Yin:** Conceptualization, Supervision, Resources, Project administration, Writing – review & editing.

## Declaration of competing interest

The authors declare no competing interests.

## Data Availability

Data will be made available on request.
